# Supplementary material for: CD8+ T cell priming that is required for curative intratumorally anchored anti-4-1BB immunotherapy is constrained by Tregs
Source: Nat Commun. 2024 Mar 1;15:1900. doi: 10.1038/s41467-024-45625-0 (PMC10907589; doi:10.1038/s41467-024-45625-0)
Supplement: Supplementary file 1 — Supplementary Information [file 41467_2024_45625_MOESM1_ESM.pdf]

Tregs constrain CD8+ T cell priming required for curative intratumorally anchored anti-4-1BB immunotherapy

Supplementary Information

Joseph R. Palmeri[1,2], Brianna M. Lax[1,2] , Joshua M. Peters[3,4], Lauren Duhamel[1,3], Jordan A. Stinson[1,3], Luciano Santollani[1,2], Emi A. Lutz[1,3], William Pinney III[1,3], Bryan D. Bryson[3,4], K. Dane Wittrup

[1]Koch Institute for Integrative Cancer Research, Departments of [2]Chemical Engineering and [3]Biological Engineering of Massachusetts Institute of Technology (MIT), [4]Ragon Institute of MIT, MGH, and Harvard

[*]corresponding author (wittrup@mit.edu)

Inventory of Supplementary Information

Supplementary Figure 1: α4-1BB-LAIR behaves as expected in vitro and in vivo

Supplementary Figure 2: Monotherapies are not efficacious and Tx + αCD4 exhibits no toxicity

Supplementary Figure 3: Tx + αCD4 also displays improved efficacy over individual components in MC38 model

Supplementary Figure 4: Tumor supernatant cytokine/chemokine analysis does not explain differences in efficacy

Supplementary Figure 5: Balance between stem-like and terminally differentiated $CD8^+$ T cells unchanged

Supplementary Figure 6: 4-1BB expression on $CD8^+$ T cells uniform across treatment groups

Supplementary Figure 7: Gene clusters exhibit differential expression among various experimental cohorts

Supplementary Figure 8: Low dose 75 ng (i.t.) DT depletes Tregs to a similar extent as 125 ng (i.t.)

Supplementary Figure 9: Delayed FTY720 initiation does not affect therapeutic efficacy of Tx + αCD4

Supplementary Figure 10: Example gating

Supplementary Figure 11: Low read samples removed from RNA-sequencing analysis

Supplementary Table 1: Amino Acid Sequences

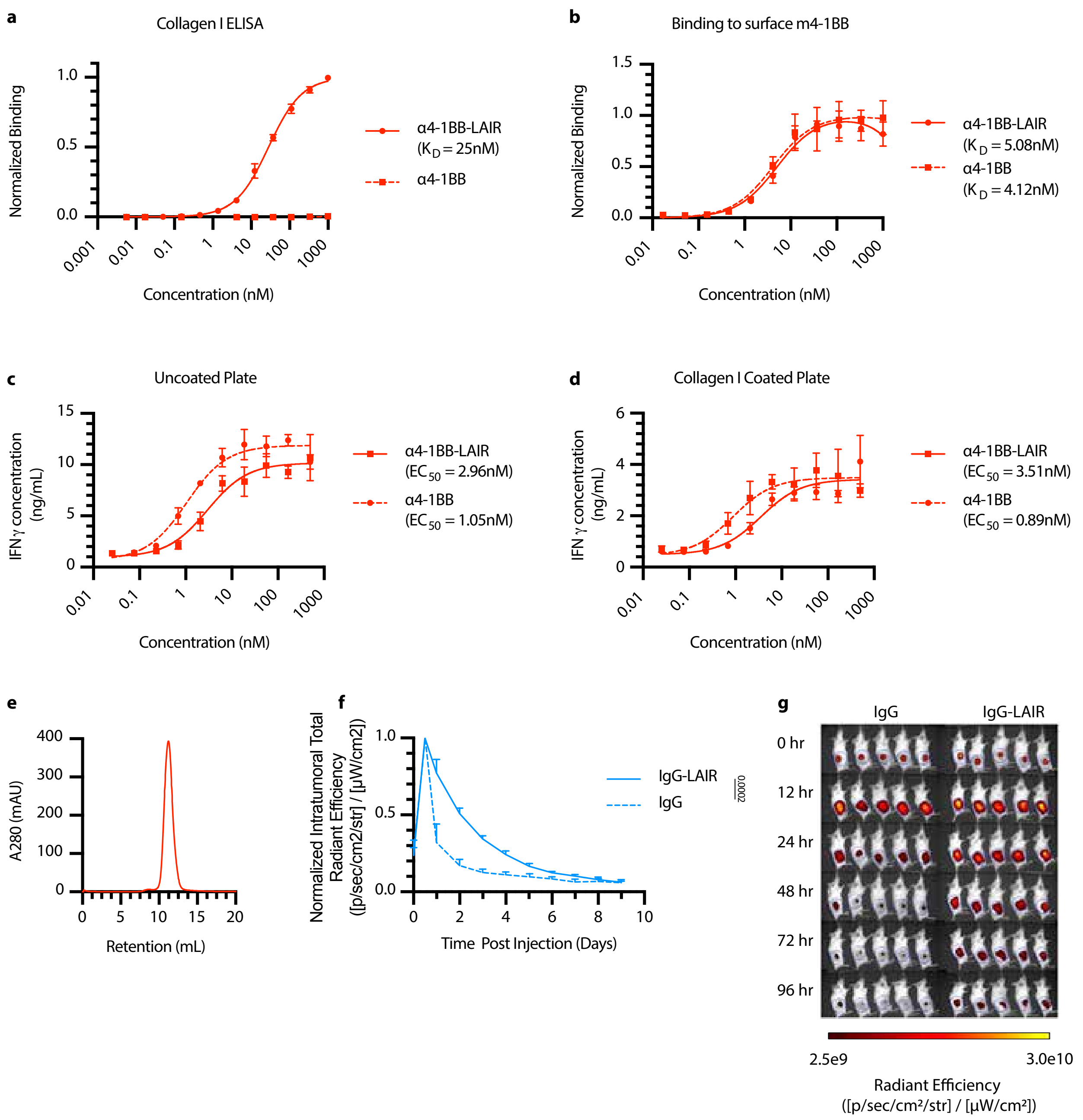

Supplementary Figure 1. α4-1BB-LAIR behaves as expected *in vitro* and *in vivo*

a, Equilibrium binding curve of α4-1BB-LAIR and α4-1BB to collagen I coated plates (mean ± S.D., n = 4). **b,** Equilibrium binding curve of α4-1BB-LAIR and α4-1BB to HEK cells expressing murine 4-1BB (mean ± S.D., n = 4). **c-d,** OT-1 splenocytes pulsed with G4 OVA peptide variant were incubated with indicated concentrations of α4-1BB and α4-1BB-LAIR on either **(c)** uncoated or **(d)** collagen I coated flat bottom plates for 72 hours and IFNγ production was measured via ELISA (mean ± S.D., n = 3). (E) SEC chromatogram of α4-1BB-LAIR. **f-g,** Mice were

inoculated with 1 x 10^6 B16F10-Trp2 KO cells on day 0, injected with 20 μg of fluorescently labeled control IgG or equimolar amount of IgG-LAIR, and fluorescence was measured longitudinally via IVIS. **f,** example fluorescence images from select timepoints and **g,** Quantification of normalized radiant efficiency (mean ± S.D.) in mice receiving IgG or IgG-LAIR (n = 5). Retention data were compared using two-way ANOVA with Tukey's multiple hypothesis testing correction. "n.s." = not significant ($P > 0.05$).

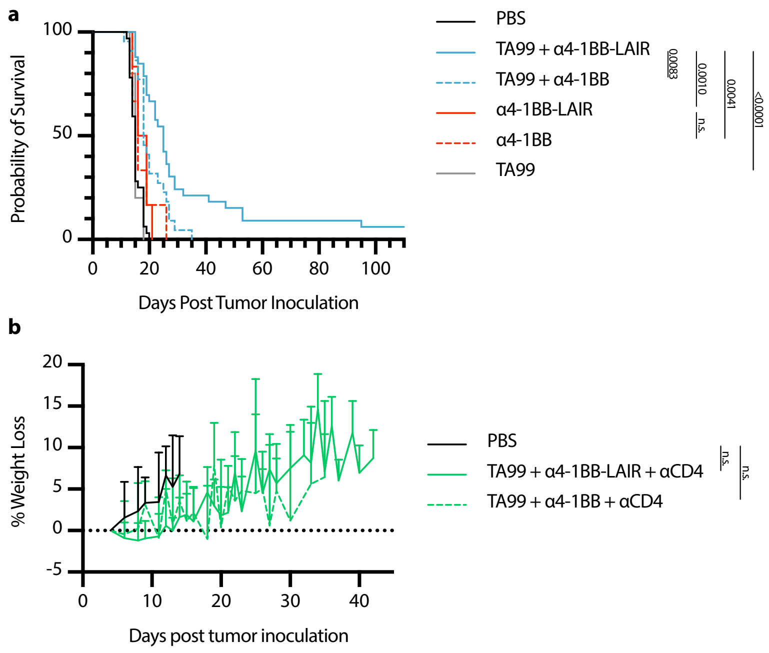

Supplementary Figure 2. Monotherapies are not efficacious and Tx + αCD4 exhibits no toxicity

Mice were inoculated with 1 x 10^6 B16F10 cells on day 0. **a,** Overall survival of mice treated with PBS (n = 32), TA99 + α4-1BB-LAIR ("Tx", n = 33), TA99 + α4-1BB (n = 22), TA99 (n = 5), α4-1BB (n = 6), or α4-1BB-LAIR (n = 6) with treatment schedule outlined in Fig. 1a (six independent studies). **b,** Weight loss of mice treated with PBS (n = 10), TA99 + α4-1BB-LAIR + αCD4 (n = 10), or TA99 + α4-1BB + αCD4 (n = 9) from survival study shown in Fig. 1D (two independent studies). Survival was compared using the log-rank Mantel-Cox test and weight loss data were compared using two-way ANOVA with Tukey's multiple hypothesis testing correction. "n.s." = not significant ($P > 0.05$).

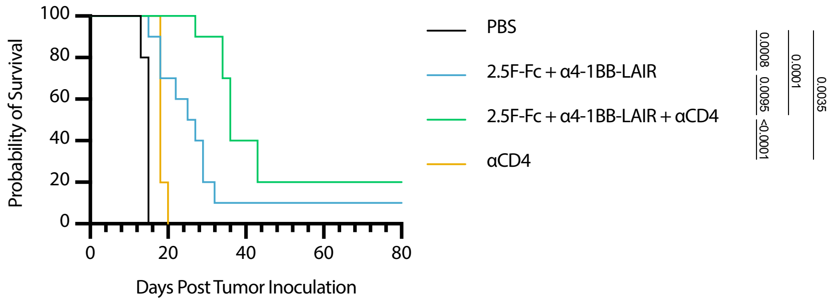

Supplementary Figure 3. Tx + αCD4 also displays improved efficacy over individual components in MC38 model

Mice were inoculated with 1 x 10^6 MC38 cells on day 0. Overall survival of mice treated with PBS (n = 5), 2.5F-Fc + α4-1BB-LAIR (n = 10), αCD4 (n = 5), or 2.5F-Fc + α4-1BB-LAIR + αCD4 (n = 10) with treatment schedule outlined in Fig. 1A. Survival was compared using the log-rank Mantel-Cox test and weight loss data were compared using two-way ANOVA with Tukey's multiple hypothesis testing correction. "n.s." = not significant ($P > 0.05$).

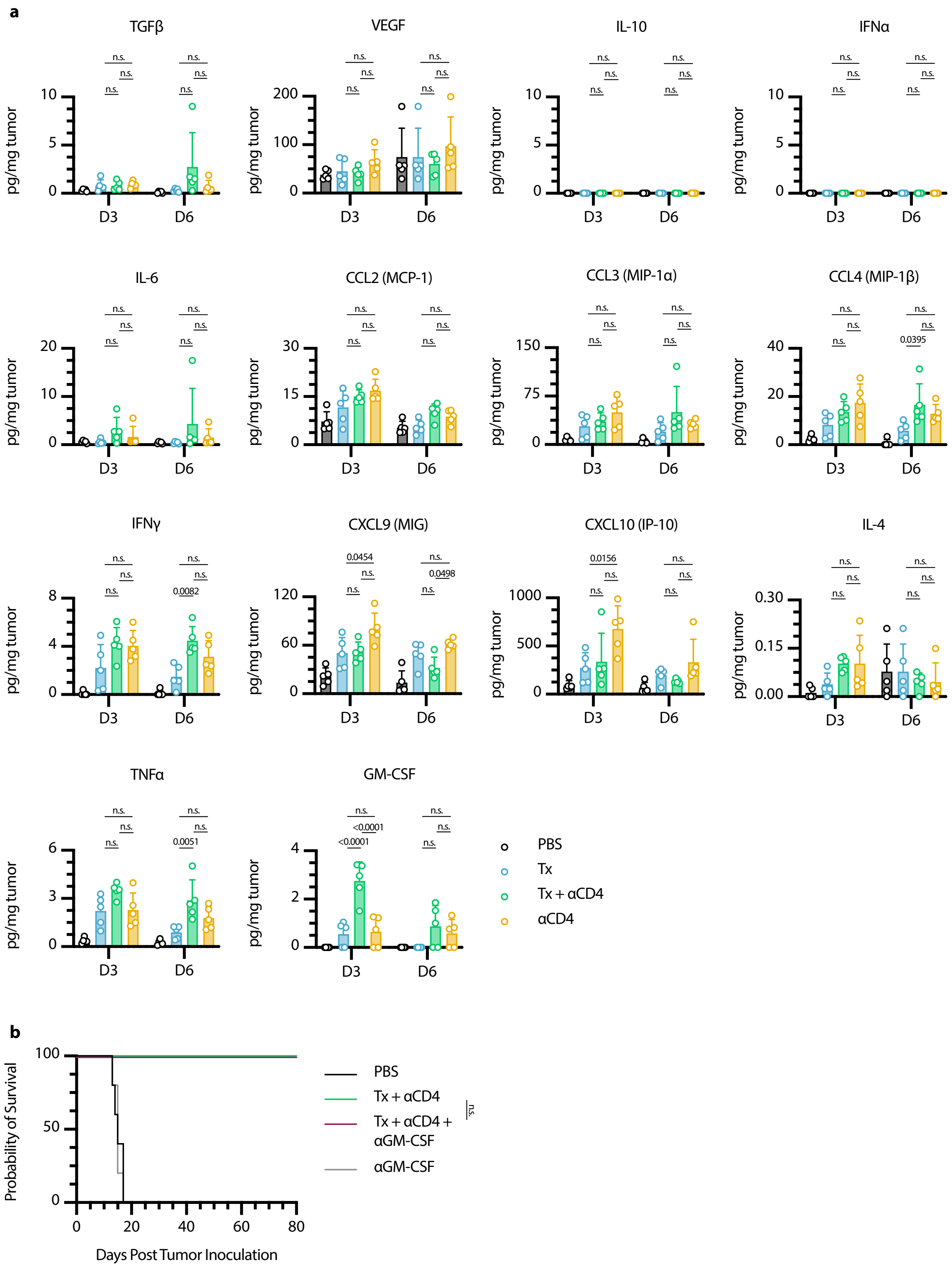
a
TGFβ
VEGF
IL-10
IFNα
IL-6
CCL2 (MCP-1)
CCL3 (MIP-1α)
CCL4 (MIP-1β)
IFNγ
CXCL9 (MIG)
CXCL10 (IP-10)
IL-4
TNFα
GM-CSF
pg/mg tumor
D3
D6
n.s.
0.0395
0.0454
0.0498
0.0156
0.0082
0.0051
<0.0001
PBS
Tx
Tx + αCD4
αCD4
b
Probability of Survival
Days Post Tumor Inoculation
PBS
Tx + αCD4
Tx + αCD4 + αGM-CSF
αGM-CSF
n.s.

Supplementary Figure 4. Tumor supernatant cytokine/chemokine analysis does not explain differences in efficacy

a, Measured levels of indicated soluble cytokines/chemokines in tumor supernatant 3 and 6 days after first α4-1BB-LAIR treatment (n = 5). **b,** Survival of mice treated with PBS (n = 5), Tx + αCD4 (n = 7), Tx + αCD4 + αGM-CSF (n = 7), or αGM-CSF (n = 5). Chemokine/cytokine measurements were compared using two-way ANOVA with Tukey's multiple hypothesis testing correction. Survival was compared using the log-rank Mantel-Cox test. "n.s." = not significant ($P > 0.05$).

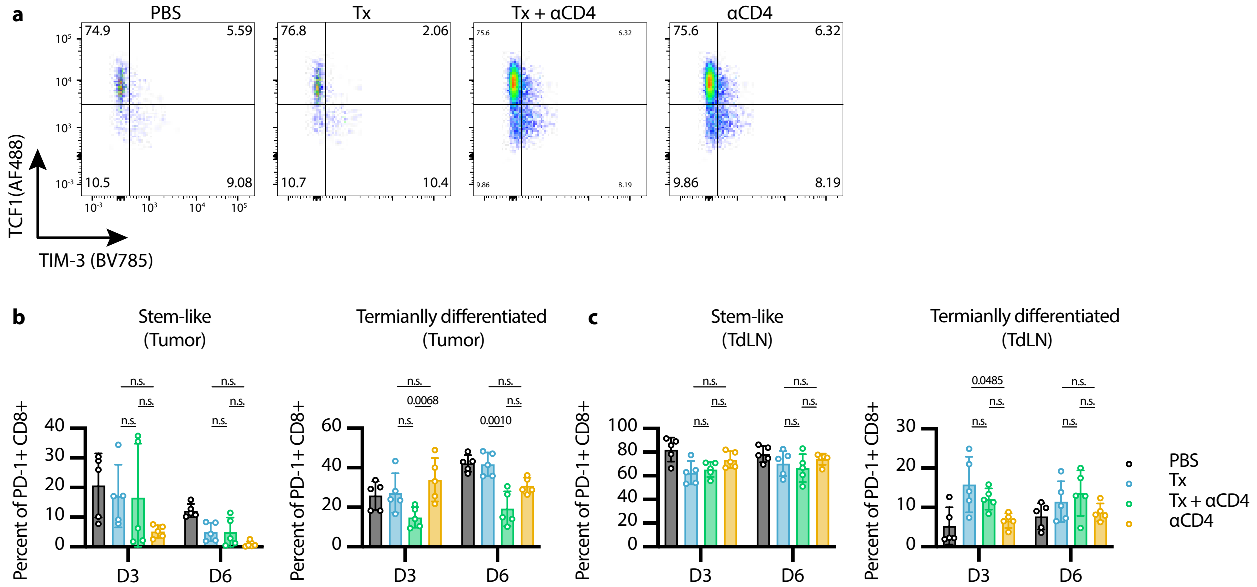

Supplementary Figure 5. Balance between stem-like and terminally differentiated CD8⁺ T cells unchanged

a, Representative gating of TCF1 and TIM-3 on PD-1⁺ CD8⁺ T cells in the TdLN 6 days after first α4-1BB-LAIR treatment. **b-c,** Flow cytometry quantification (mean±SD) of stem-like (TCF1⁺ TIM-3⁻) and terminally differentiated (TCF⁻ TIM-3⁺) PD-1⁺ CD8⁺ T cells in the **(b)** tumor and **(c)** TdLN 3 and 6 days after first α4-1BB-LAIR treatment (gated on single cell/live/CD45⁺/CD3⁺NK1.1⁻/CD8⁺/PD-1⁺, n = 5). Flow data were compared using two-way ANOVA with Tukey's multiple hypothesis testing correction. "n.s." = not significant (*P* > 0.05).

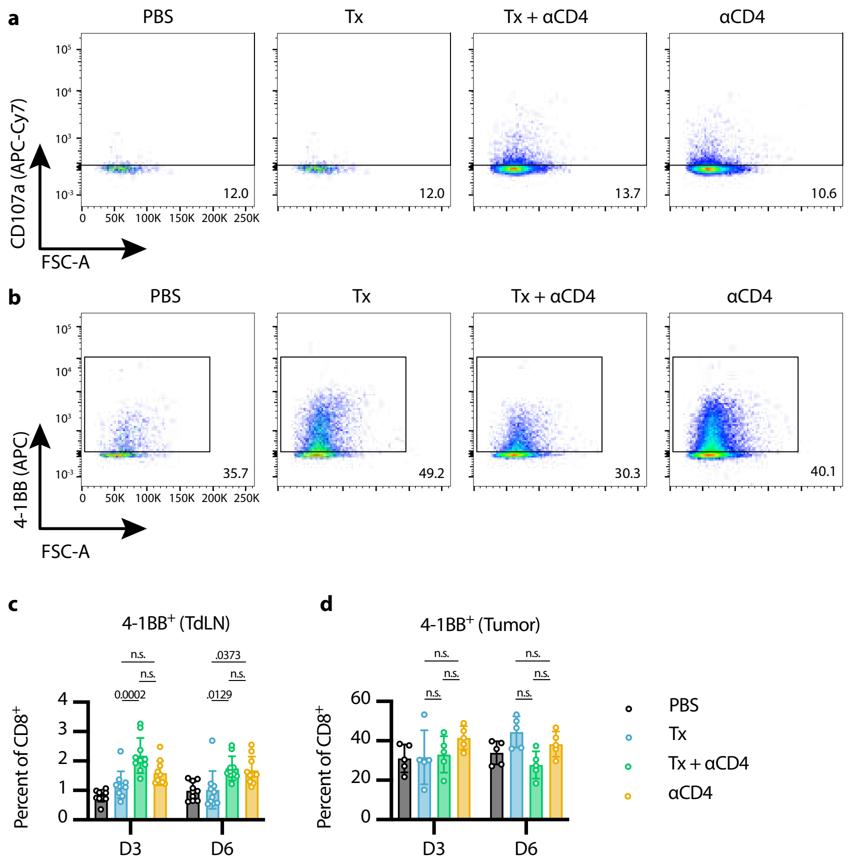

Supplementary Figure 6. 4-1BB expression on CD8+ T cells uniform across treatment groups

a, Representative gating of CD107a+ CD8+ T cells in the tumor 6 days after first α4-1BB-LAIR treatment. **b,** Representative gating of 4-1BB+ CD8+ T cells in the tumor 6 days after first α4-1BB-LAIR treatment. **c-d,** Flow cytometry quantification (mean±SD) of 4-1BB+ CD8+ T cells in the **(c)** TdLN and **(d)** tumor 3 and 6 days after first α4-1BB-LAIR treatment (gated on single cell/live/CD45+/CD3+NK1.1-/CD8+, n = 5-10, two independent experiments). Flow data were compared using two-way ANOVA with Tukey's multiple hypothesis testing correction. "n.s." = not significant ($P > 0.05$).

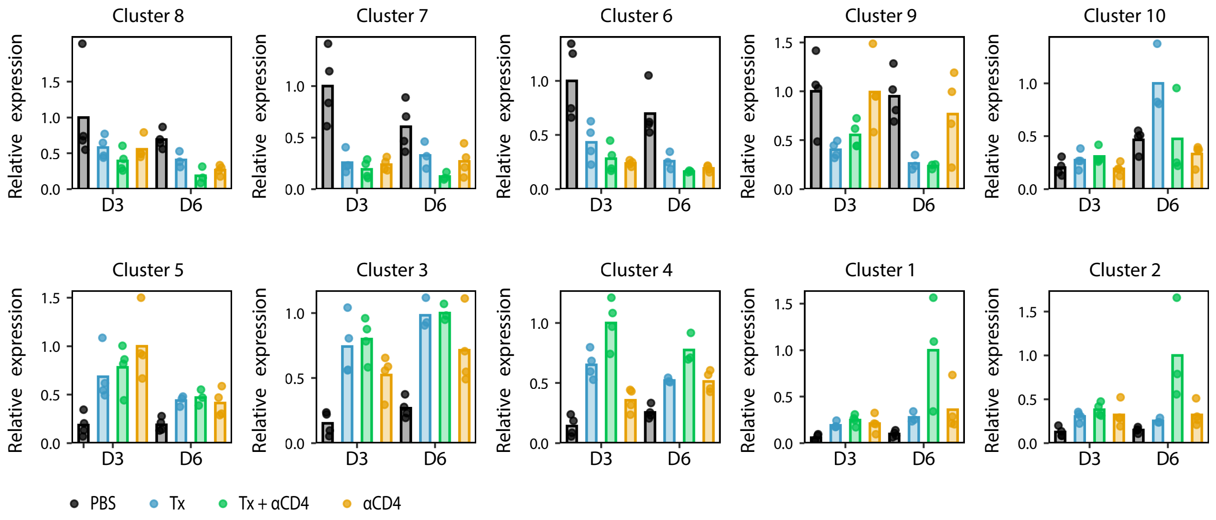

Supplementary Figure 7. Gene clusters exhibit differential expression among various experimental cohorts

Normalized expression of all gene clusters identified in Fig. 4a for different experimental cohorts.

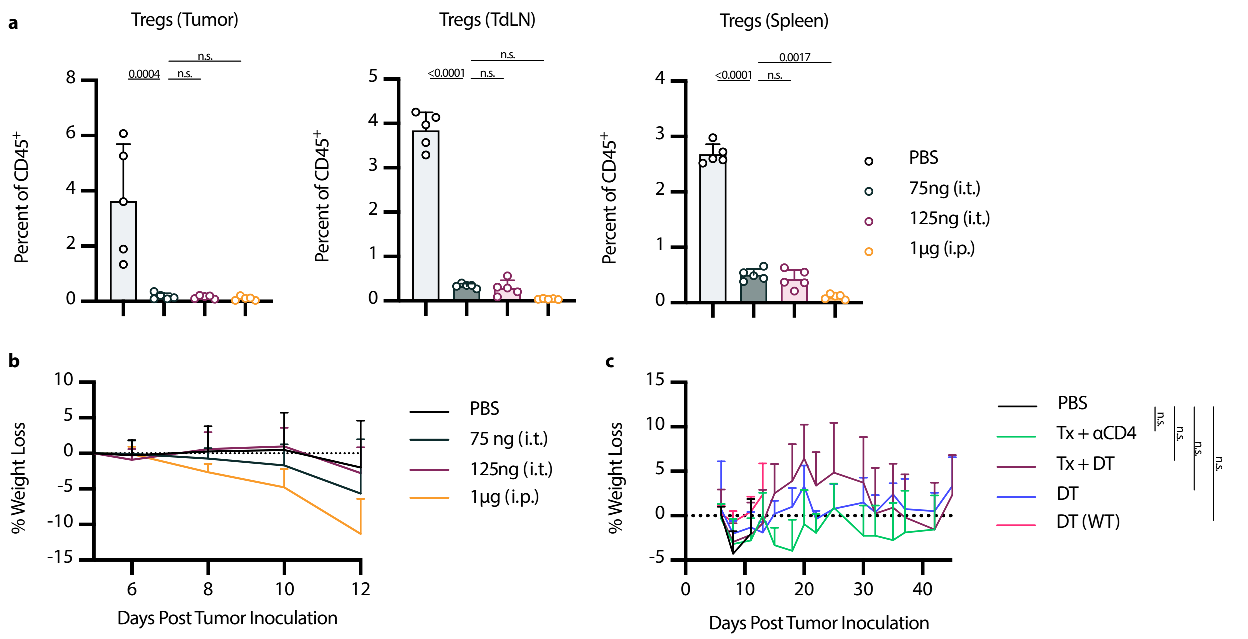

Supplementary Figure 8. Low dose 75 ng (i.t.) DT depletes Tregs to a similar extent as 125 ng (i.t.)

Foxp3-DTR Mice were inoculated with 1 x 10^6 B16F10 cells on day 0. **a,** Mice were treated on days 6, 8, and 10 with either 125 ng DT (i.t.), 75 ng DT (i.t.), or 1 μg DT (i.p.). Flow cytometry quantification (mean±SD) of Tregs in tumor, TdLN, or spleen on day 12 (gated on single cell/live/$CD45^+$/$CD3^+$NK1.1^-/$CD4^+$/GFP(*Foxp3*)$^+$, n = 5). **b,** Weight loss (mean+SD) of mice from **a**. **c,** Weight loss data from survival study shown in Fig. 5c. Flow data were compared using one-way ANOVA with Tukey's multiple hypothesis testing correction and weight loss data were compared using two-way ANOVA with Tukey's multiple hypothesis testing correction. "n.s." = not significant ($P > 0.05$).

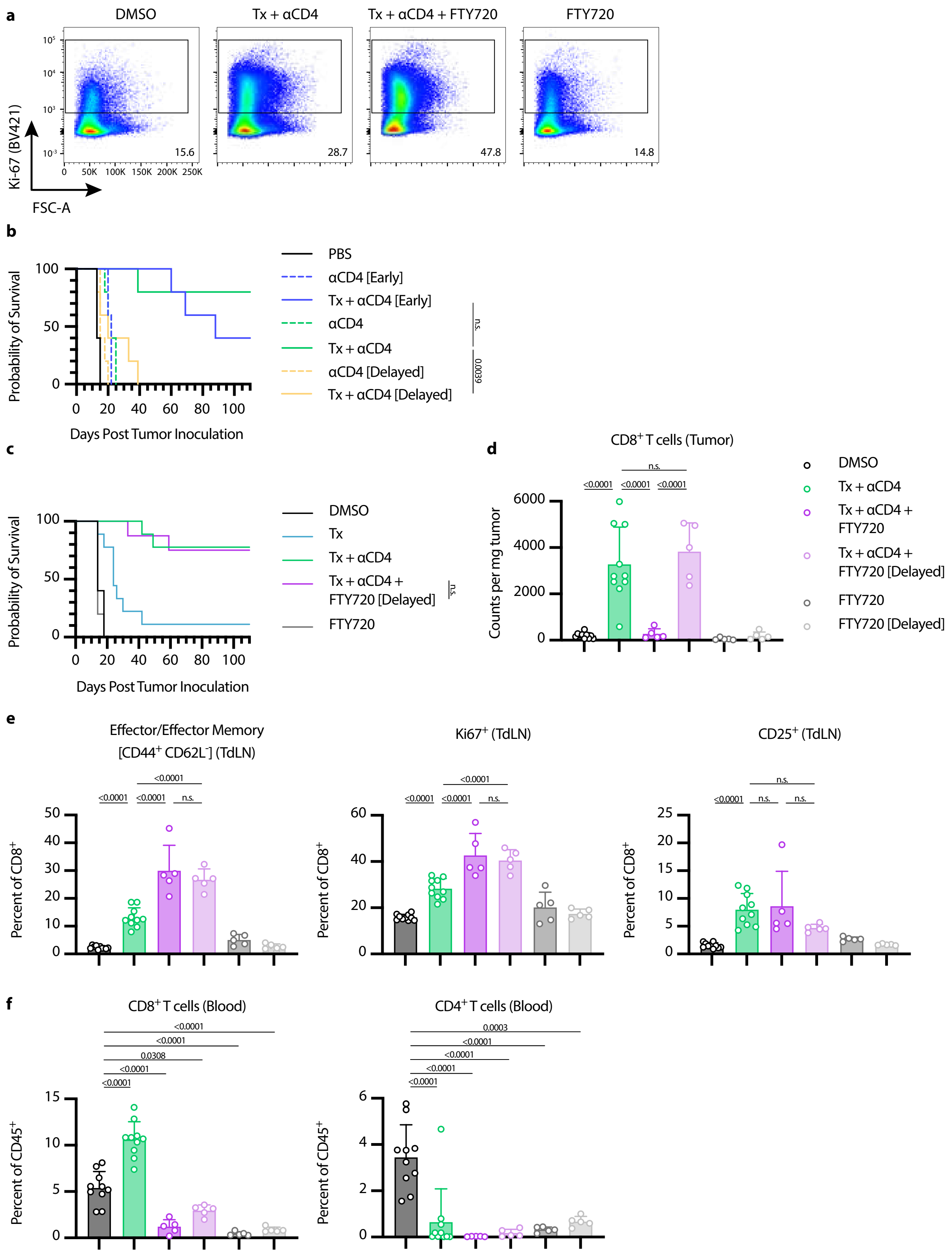
a
DMSO
Tx + αCD4
Tx + αCD4 + FTY720
FTY720
Ki-67 (BV421)
FSC-A
15.6
28.7
47.8
14.8
b
Probability of Survival
Days Post Tumor Inoculation
PBS
αCD4 [Early]
Tx + αCD4 [Early]
αCD4
Tx + αCD4
αCD4 [Delayed]
Tx + αCD4 [Delayed]
n.s.
0.0039
c
Probability of Survival
Days Post Tumor Inoculation
DMSO
Tx
Tx + αCD4
Tx + αCD4 + FTY720 [Delayed]
FTY720
n.s.
d
CD8+ T cells (Tumor)
Counts per mg tumor
DMSO
Tx + αCD4
Tx + αCD4 + FTY720
Tx + αCD4 + FTY720 [Delayed]
FTY720
FTY720 [Delayed]
e
Effector/Effector Memory [CD44+ CD62L-] (TdLN)
Ki67+ (TdLN)
CD25+ (TdLN)
Percent of CD8+
f
CD8+ T cells (Blood)
CD4+ T cells (Blood)
Percent of CD45+

Supplementary Figure 9. Delayed FTY720 initiation does not affect therapeutic efficacy of Tx + αCD4

a, Representative gating for Ki67$^+$ CD8$^+$ T cells in TdLN 6 days after first α4-1BB-LAIR treatment (gated on single cell/live/CD45$^+$/CD3$^+$NK1.1$^-$/CD8$^+$). **b,** Overall Survival of mice treated with PBS, Tx + αCD4, or αCD4 with αCD4 initiated on day 4 as outlined in fig. 1A, day 10 ("delayed"), or day -8 ("early") (n = 5). **c-f,** Delayed FTY720 refers to FTY720 initiation concurrent with α4-1BB-LAIR treatment, while FTY720 refers to FTY720 initiation concurrent with αCD4. **c,** Overall survival of mice treated with PBS/DMSO (n = 5), Tx (n = 9), Tx + αCD4 (n = 9), Tx + αCD4 + delayed FTY720 (n = 8), or delayed FTY720 (n = 5). Mice were treated with the same dose/dose schedule as in Fig 1a, with delayed FTY720 treatment initiated on day 6 and continued every other day until day 34. **d,** Flow cytometry quantification (mean±SD) of CD8$^+$ T cells in the tumor 6 days after first α4-1BB-LAIR treatment (gated on single cell/live/CD45$^+$/CD3$^+$NK1.1$^-$/CD8$^+$, n = 5-10, two independent experiments). **e,** Flow cytometry quantification (mean±SD) of effector/effector memory (CD44$^+$ CD62L$^-$), CD25$^+$, and Ki67$^+$ CD8$^+$ T cells in the TdLN 6 days after first α4-1BB-LAIR treatment (gated on single cell/live/CD45$^+$/CD3$^+$NK1.1$^-$/CD8$^+$, n = 5-10, two independent experiments). **f,** Flow cytometry quantification (mean±SD) of CD8$^+$ T cells and CD4$^+$ T cells in the blood 6 days after first α4-1BB-LAIR treatment (gated on single cell/live/CD45$^+$/CD3$^+$NK1.1$^-$/CD8$^+$, n = 5-10, two independent experiments). Survival data were compared using log-rank Mantel-Cox test and Flow cytometry data were compared using one-way ANOVA with Tukey's multiple hypothesis testing correction. "n.s." = not significant ($P > 0.05$).

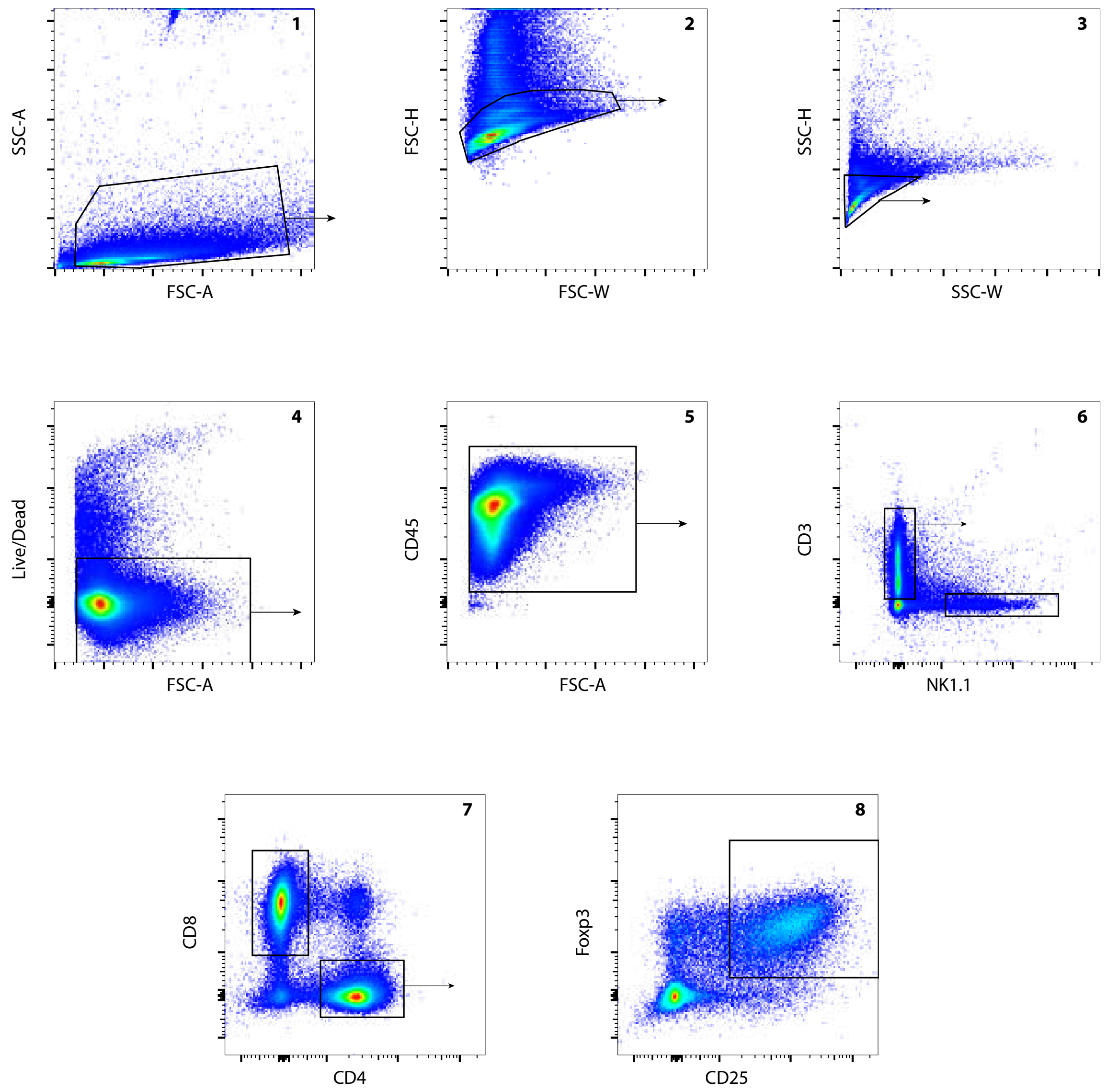

Supplementary Figure 10. Example gating

Gating strategy for $CD8^+$ T cells, $CD4^+$ T cells, and $Foxp3^+$ $CD25^+$ Tregs, shown on a TdLN sample. Identical gating strategies were used for tumor, spleen, and blood samples.

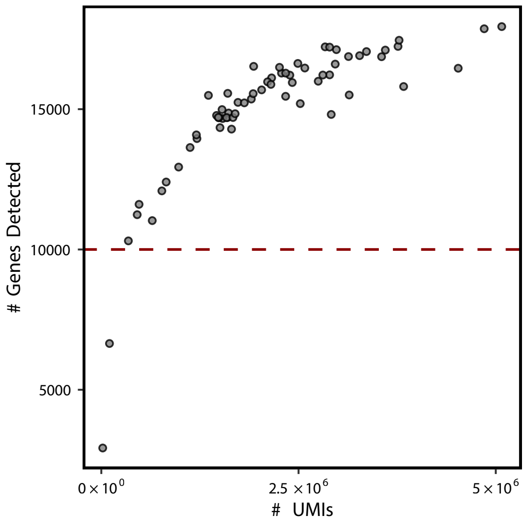

Supplementary Figure 11. Low read samples removed from RNA-sequencing analysis

Plot of number of genes detected versus number of unique reads per sample for all tumor and TdLN bulk-RNA seq samples. Samples with less than 10,000 unique genes detected were excluded from analysis. Two tumor samples (one Tx D6 and one Tx + αCD4 D6) met this exclusion criteria.

Table S1: Amino Acid Sequences

Key: signal peptide, variable region, constant region, linker, LAIR

α4-1BB Light Chain (murine kappa constant region)	MSVLTQVLALLLLWLTGARCADIQMTQSPASLSASLEEIVTITCQASQDIGN WLAWYHQKPGKSPQLLIYGSTSLADGVPSRFSGSSSGSQYSLKISRLQVEDIG IYYCLQAYGAPWTFGGGTKLELKRADAAPTVSIFPPSSEQLTSGGASVVCFLN NFYPKDINVKWKIDGSERQNGVLNSWTDQDSKDSTYSMSSTLTLTKDEYER HNSYTCEATHKTSTSPIVKSFNRNEC
α4-1BB Heavy Chain (murine IgG1 constant region)	MKWSWVFLFLMAMVTGVNSDVQLVESGGGLVQPGRSLKLSCAASGFIFSY FDMAWVRQAPTKGLEWVASISPDGSIPYYRDSVKGRFTVSRENAKSSLYLQ MDSLRSEDTATYYCARRSYGGYSEIDYWGQGVMVTVSSATTKGPSVYPLAP GSAAQTNSMVTLGCLVKGYFPEPVTVTWNSGSLSSGVHTFPAVLQSDLYTL SSSVTVPSSTWPSQTVTCNVAHPASSTKVDKKIVPRDCGCKPCICTVPEVSSV FIFPPKPKDVLTITLTPKVTCVVVDISKDDPEVQFSWFVDDVEVHTAQTKPREE QINSTFRSVSELPIMHQDWLNGKEFKCRVNSAAFPAPIEKTISKTKGRPKAPQ VYTIPPPKEQMAKDKVSLTCMITNFFPEDITVEWQWNGQPAENYKNTQPIM DTDGSYFVYSKLNVQKSNWEAGNTFTCSVLHEGLHNHHTEKSLSHSPGK
α4-1BB-LAIR Heavy Chain (murine IgG1 constant region)	MKWSWVFLFLMAMVTGVNSDVQLVESGGGLVQPGRSLKLSCAASGFIFSY FDMAWVRQAPTKGLEWVASISPDGSIPYYRDSVKGRFTVSRENAKSSLYLQ MDSLRSEDTATYYCARRSYGGYSEIDYWGQGVMVTVSSATTKGPSVYPLAP GSAAQTNSMVTLGCLVKGYFPEPVTVTWNSGSLSSGVHTFPAVLQSDLYTL SSSVTVPSSTWPSQTVTCNVAHPASSTKVDKKIVPRDCGCKPCICTVPEVSSV FIFPPKPKDVLTITLTPKVTCVVVDISKDDPEVQFSWFVDDVEVHTAQTKPREE QINSTFRSVSELPIMHQDWLNGKEFKCRVNSAAFPAPIEKTISKTKGRPKAPQ VYTIPPPKEQMAKDKVSLTCMITNFFPEDITVEWQWNGQPAENYKNTQPIM DTDGSYFVYSKLNVQKSNWEAGNTFTCSVLHEGLHNHHTEKSLSHSPGKGG GGSGGGGSGGGGSQEGSLPDITIFPNSSLMISQGTFVTVVCSYSDKHDLYN MVRLEKDGSTFMEKSTEPYKTEDEFEIGPVNETITGHYSCIYSKGITWSERSKT LELKVIKENVIQTPAPGPTSDTSWLKTYSIY
αFITC Light Chain (murine kappa constant region)	MSVLTQVLALLLLWLTGARCADVVMTQTPLSLPVSLGDQASISCRSSQSLVH SNGNTYLRWYLQKPGQSPKVLIYKVSNRFSGVPDRFSGSGSGTDFTLKISRVE AEDLGVYFCSQSTHVPWTFGGGTKLEIKRADAAPTVSIFPPSSEQLTSGGASV VCFLNNFYPKDINVKWKIDGSERQNGVLNSWTDQDSKDSTYSMSSTLTLTK DEYERHNSYTCEATHKTSTSPIVKSFNRNEC

αFITC Heavy Chain (murine IgG2c constant region, **LALA-PG silencing mutations bolded and underlined**)	MKWSWVFLFLMAMVTGVNSDVKLDETGGGLVQPGRPMKLSCVASGFTFSDYWMNWVRQSPEKGLEWVAQIRNKPYNYETYYSDSVKGRFTISRDDSKSSVYLQMNNLRVEDMGIYYCTGSYYGMDYWGQGTSVTVSAKTTAPSVYPLAPVCGDTTGSSVTLGCLVKGYFPEPVTLTWNSGSLSSGVHTFPAVLQSDLYTLSSSVTVTSSTWPSQSITCNVAHPASSTKVDKKIEPRGPTIKPCPPCKCPAPN**AA**GGPSVFIFPPKIKDVLMISLSPIVTCVVVDVSEDDPDVQISWFVNNVEVHTAQTQTHREDYNSTLRVVSALPIQHQDWMSGKEFKCKVNNKDL**G**APIERTISKPKGSVRAPQVYVLPPPEEEMTKKQVTLTCMVTDFMPEDIYVEWTNNGKTELNYKNTEPVLDSDGSYFMYSKLRVEKKNWVERNSYSCSVVHEGLHNHHTTKSFSRTPGK
αFITC-LAIR Heavy Chain (murine IgG2c constant region, **LALA-PG silencing mutations bolded and underlined**)	MKWSWVFLFLMAMVTGVNSDVKLDETGGGLVQPGRPMKLSCVASGFTFSDYWMNWVRQSPEKGLEWVAQIRNKPYNYETYYSDSVKGRFTISRDDSKSSVYLQMNNLRVEDMGIYYCTGSYYGMDYWGQGTSVTVSAKTTAPSVYPLAPVCGDTTGSSVTLGCLVKGYFPEPVTLTWNSGSLSSGVHTFPAVLQSDLYTLSSSVTVTSSTWPSQSITCNVAHPASSTKVDKKIEPRGPTIKPCPPCKCPAPN**AA**GGPSVFIFPPKIKDVLMISLSPIVTCVVVDVSEDDPDVQISWFVNNVEVHTAQTQTHREDYNSTLRVVSALPIQHQDWMSGKEFKCKVNNKDL**G**APIERTISKPKGSVRAPQVYVLPPPEEEMTKKQVTLTCMVTDFMPEDIYVEWTNNGKTELNYKNTEPVLDSDGSYFMYSKLRVEKKNWVERNSYSCSVVHEGLHNHHTTKSFSRTPGKGGGGSGGGGSGGGGSQEGSLPDITIFPNSSLMISQGTFVTVVCSYSDKHDLYNMVRLEKDGSTFMEKSTEPYKTEDEFEIGPVNETITGHYSCIYSKGITWSERSKTLELKVIKENVIQTPAPGPTSDTSWLKTYSIY
TA99 Light Chain (murine kappa constant region)	MSVLTQVLALLLLWLTGARCAIQMSQSPASLSASVGETVTITCRASGNIYNYLAWYQQKQGKSPHLLVYDAKTLADGVPSRFSGSGSGTQYSLKISSLQTEDSGNYYCQHFWSLPFTFGSGTKLEIKRADAAPTVSIFPPSSEQLTSGGASVVCFLNNFYPKDINVKWKIDGSERQNGVLNSWTDQDSKDSTYSMSSTLTLTKDEYERHNSYTCEATHKTSTSPIVKSFNRNEC
TA99 Heavy Chain (murine IgG2c constant region)	MKWSWVFLFLMAMVTGVNSEVQLQQSGAELVRPGALVKLSCKTSGFNIKDYFLHWVRQRPDQGLEWIGWINPDNGNTVYDPKFQGTASLTADTSSNTVYLQLSGLTSEDTAVYFCTRRDYTYEKAALDYWGQGASVIVSSAKTTAPSVYPLAPVCGGTTGSSVTLGCLVKGYFPEPVTLTWNSGSLSSGVHTFPALLQSGLYTLSSSVTVTSNTWPSQTITCNVAHPASSTKVDKKIEPRVPITQNPCPPLKECPPCAAPDLLGGPSVFIFPPKIKDVLMISLSPMVTCVVVDVSEDDPDVQISWFVNNVEVHTAQTQTHREDYNSTLRVVSALPIQHQDWMSGKEFKCKVNNRALPSPIEKTISKPRGPVRAPQVYVLPPPAEEMTKKEFSLTCMITGFLPAEIAVDWTSNGRTEQNYKNTATVLDSDGSYFMYSKLRVQKSTWERGSLFACSVVHEGLHNHLTTKTISRSLGK
2.5F-Fc (murine IgG2c constant region)	MRVPAQLLGLLLLWLPGARCGCPRPRGDNPPLTCSQDSDCLAGCVCGPNGFCGGRLEPRVPITQNPCPPLKECPPCAAPDLLGGPSVFIFPPKIKDVLMISLSPMVTCVVVDVSEDDPDVQISWFVNNVEVHTAQTQTHREDYNSTLRVVSALPIQHQDWMSGKEFKCKVNNRALPSPIEKTISKPRGPVRAPQVYVLPPPAEEMTKKEFSLTCMITGFLPAEIAVDWTSNGRTEQNYKNTATVLDSDGSYFMYSKLRVQKSTWERGSLFACSVVHEGLHNHLTTKTISRSLGK

9D9 Light Chain (murine kappa constant region)	MDMRVPAQLLGLLLLWLPGARCDIVMTQTTLSLPVSLGDQASISCRSSQSIV<br>HSNGNTYLEWYLQKPGQSPKLLIYKVSNRFSGVPDRFSGSGSGTDFTLKISRV<br>EAEDLGVYYCFQGSHVPYTFGGGTKLEIKRADAAPTVSIFPPSSEQLTSGGAS<br>VVCFLNNFYPKDINVKWKIDGSERQNGVLNSWTDQDSKDSTYSMSSTLTLT<br>KDEYERHNSYTCEATHKTSTSPIVKSFNRNEC
9D9 Heavy Chain (murine IgG2c constant region)	MGWSLILLFLVAVATGVHSEAKLQESGPVLVKPGASVKMSCKASGYTFTDYY<br>MNWVKQSHGKSLEWIGVINPYNGDTSYNQKFKGKATLTVDKSSSTAYMEL<br>NSLTSEDSAVYYCARYYGSWFAYWGQGTLITVSTAKTTAPSVYPLAPVCGGT<br>TGSSVTLGCLVKGYFPEPVTLTWNSGSLSSGVHTFPALLQSGLYTLSSSVTVT<br>SNTWPSQTITCNVAHPASSTKVDKKIEPRVPITQNPCPPLKECPPCAAPDLLG<br>GPSVFIFPPKIKDVLMISLSPMVTCVVVDVSEDDPDVQISWFVNNVEVHTAQ<br>TQTHREDYNSTLRVVSALPIQHQDWMSGKEFKCKVNNRALPSPIEKTISKPR<br>GPVRAPQVYVLPPPAEEMTKKEFSLTCMITGFLPAEIAVDWTSNGRTEQNYK<br>NTATVLDSDGSYFMYSKLRVQKSTWERGSLFACSVVHEGLHNHLTTKTISRS<br>LGK
