# Supplementary material for: CD8+ T cell priming that is required for curative intratumorally anchored anti-4-1BB immunotherapy is constrained by Tregs
Source: Nat Commun. 2024 Mar 1;15:1900. doi: 10.1038/s41467-024-45625-0 (PMC10907589; doi:10.1038/s41467-024-45625-0)
Supplement: Supplementary file 3 — Reporting Summary [file 41467_2024_45625_MOESM3_ESM.pdf]

nature portfolio

Corresponding author(s): K. Dane Wittrup

Last updated by author(s): Oct 26, 2023

Reporting Summary

Nature Portfolio wishes to improve the reproducibility of the work that we publish. This form provides structure for consistency and transparency in reporting. For further information on Nature Portfolio policies, see our Editorial Policies and the Editorial Policy Checklist.

Statistics

For all statistical analyses, confirm that the following items are present in the figure legend, table legend, main text, or Methods section.

n/a	Confirmed	
☐	☒	The exact sample size (*n*) for each experimental group/condition, given as a discrete number and unit of measurement
☐	☒	A statement on whether measurements were taken from distinct samples or whether the same sample was measured repeatedly
☐	☒	The statistical test(s) used AND whether they are one- or two-sided<br>*Only common tests should be described solely by name; describe more complex techniques in the Methods section.*
☒	☐	A description of all covariates tested
☐	☒	A description of any assumptions or corrections, such as tests of normality and adjustment for multiple comparisons
☐	☒	A full description of the statistical parameters including central tendency (e.g. means) or other basic estimates (e.g. regression coefficient) AND variation (e.g. standard deviation) or associated estimates of uncertainty (e.g. confidence intervals)
☐	☒	For null hypothesis testing, the test statistic (e.g. *F*, *t*, *r*) with confidence intervals, effect sizes, degrees of freedom and *P* value noted<br>*Give P values as exact values whenever suitable.*
☒	☐	For Bayesian analysis, information on the choice of priors and Markov chain Monte Carlo settings
☒	☐	For hierarchical and complex designs, identification of the appropriate level for tests and full reporting of outcomes
☒	☐	Estimates of effect sizes (e.g. Cohen's *d*, Pearson's *r*), indicating how they were calculated

Our web collection on statistics for biologists contains articles on many of the points above.

Software and code

Policy information about availability of computer code

Data collection	Flow data were obtained using the BD FACSDiva software (BD Biosciences v7). ELISA plates were measured using the Tecan Infinite M200 Pro absorbance/fluorescence plate reader and software. IVIS data were collected using the IVIS Spectrum In Vivo Imaging system (Perkin-Elmer). RNA-sequencing was performed on a NextSeq 500 (Illumina).
Data analysis	Flow data were analyzed using FlowJo (v10). IVIS data were analyzed using Living Image software (Caliper Life Sciences, v4.5). Plotting and statistical analysis was performed in GraphPad Prism (v10). For RNA-sequencing data, Data preprocessing and count matrix construction were performed using the Smart-seq2 Multi-Sample v2.2.0 Pipeline (RRID:SCR_018920) on Terra. The following published R packages were used to analyze and plot sequencing data: DEseq2 (v1.34.0), Seurat (v4.1.0), complexHeatmap (v2.10.0), enrichR (v3.2), clusterProfiler (v4.2.2), ggpubr (v0.6.0). GSEA was performed using software from the Broad Institute(v4.3.2). Microsoft Excel (v16.75) was used for some data preprocessing and to compile source data files and Microsoft Word (v16.77.1) was used to write the manuscript. Adobe Illustrator (v27.8.1). See methods for additional details.

For manuscripts utilizing custom algorithms or software that are central to the research but not yet described in published literature, software must be made available to editors and reviewers. We strongly encourage code deposition in a community repository (e.g. GitHub). See the Nature Portfolio guidelines for submitting code & software for further information.

Data

Policy information about availability of data

All manuscripts must include a data availability statement. This statement should provide the following information, where applicable:

- Accession codes, unique identifiers, or web links for publicly available datasets
- A description of any restrictions on data availability
- For clinical datasets or third party data, please ensure that the statement adheres to our policy

All sequencing data generated in this study can be found in the GEO database under accession GSE223087 (https://www.ncbi.nlm.nih.gov/geo/query/acc.cgi?acc=GSE223087). All other data generated needed to evaluate conclusions in this study are available in the paper or in the figshare repository associated with this study (https://doi.org/10.6084/m9.figshare.23805444). All materials available upon request.

Research involving human participants, their data, or biological material

Policy information about studies with human participants or human data. See also policy information about sex, gender (identity/presentation), and sexual orientation and race, ethnicity and racism.

Reporting on sex and gender	N/A
Reporting on race, ethnicity, or other socially relevant groupings	N/A
Population characteristics	N/A
Recruitment	N/A
Ethics oversight	N/A

Note that full information on the approval of the study protocol must also be provided in the manuscript.

Field-specific reporting

Please select the one below that is the best fit for your research. If you are not sure, read the appropriate sections before making your selection.

☒ Life sciences ☐ Behavioural & social sciences ☐ Ecological, evolutionary & environmental sciences

For a reference copy of the document with all sections, see nature.com/documents/nr-reporting-summary-flat.pdf

Life sciences study design

All studies must disclose on these points even when the disclosure is negative.

Sample size	No statistical methods were used to determine sample sizes. Sample sizes were based on previous studies in our lab (Lax et al., Proc. Natl. Acad. Sci. USA. 120 (31), e2300895120 (2023), Lutz et al., Proc. Natl. Acad. Sci. USA. 119 (36), e2205983119 (2022), Agarwal et al., Nat. Biomed. Eng. 6, 129-143 (2022), Momin et al., Nat. Commun. 13, 109 (2022), Mehta et al., Nat. Biomed. Eng. 4, 636-648 (2020), Momin et al., Sci. Trans. Med. 11, eaaw2614 (2019)).
Data exclusions	No data was excluded from the manuscript. For RNA-sequencing analysis, samples with less than 10,000 genes detected were excluded from analysis. This led to exclusion of two tumor samples, one from the Tx + αCD4 group and one from the αCD4 group at the day 6 time point (Fig. S11). These samples are still included in dataset on GEO. This is also detailed in the methods.
Replication	In all studies there were at least 5 mice per experimental group, except for the bulk RNA-sequencing experiment which had 3-4 mice per group. Most survival experiments were repeated twice, and number of mice per group, number of experimental repeats, and statistical methods are noted in figure legends.
Randomization	Mice were randomized before treatment start to ensure uniform tumor sizes among different groups.
Blinding	Investigators were not blinded to treatment groups for logistical reasons.

Reporting for specific materials, systems and methods

We require information from authors about some types of materials, experimental systems and methods used in many studies. Here, indicate whether each material, system or method listed is relevant to your study. If you are not sure if a list item applies to your research, read the appropriate section before selecting a response.

Materials & experimental systems

n/a	Involved in the study
☐	☒ Antibodies
☐	☒ Eukaryotic cell lines
☒	☐ Palaeontology and archaeology
☐	☒ Animals and other organisms
☒	☐ Clinical data
☒	☐ Dual use research of concern
☒	☐ Plants

Methods

n/a	Involved in the study
☒	☐ ChIP-seq
☐	☒ Flow cytometry
☒	☐ MRI-based neuroimaging

Antibodies

Antibodies used

Tumor and TdLN samples in Figures 2 and S4 were stained in 100µL with anti-CD45-BUV395 (30-F11, BD Biosciences #564279, 1:100 dilution), anti-CD4-BUV563 (RM4-4, BD Biosciences #741218, 1:100 dilution), anti-CD8α-BUV737 (53-6.7 BD Biosciences #612759, 1:100 dilution), anti-CD62L-BUV805 (MEL-14, BD Biosciences #741924, 1:100 dilution), anti-CD44-BV421 (1M7, Biolegend #103040, 1:100 dilution), anti-Ki67-BV605 (16A8, Biolegend #652413, 1:100 dilution), anti-CD3-BV711 (17A2, Biolegend #100241, 1:100 dilution), anti-TIM-3-BV785 (RMT3-23, Biolegend #119725, 1:50 dilution), anti-TCF1/TCF7-AF488 (C63D9, Cell Signaling Technology #6444, 1:400 dilution), anti-PD-1-PerCp/Cy5.5 (29F.1A12, Biolegend #135208, 1:50 dilution), anti-Foxp3-PE (FJK-16s, Invitrogen #12-5773-82, 1:200 dilution), anti-CD25-PE-Cy5 (PC61, Biolegend #102010, 1:100 dilution), anti-NK1.1-PE-Cy7 (PK-136126, Biolegend #108714, 1:100 dilution), anti-4-1BB-APC (17B5, Biolegend #106110, 1:50 dilution), anti-CD107a-APC-Cy7 (1D4B, Biolegend #121616, 1:100 dilution).

Tumor, TdLN, and spleen samples in Figure 5A and S6 were stained in 100µL with anti-CD45-BUV395 (30F-11, BD Bioscience #564279, 1:100 dilution), anti-CD8a-BUV737 (53-6.7, BD Biosciences #612759, 1:100 dilution), anti-CD3-BV785 (17A2, Biolegend #100232, 1:100 dilution), anti-NK1.1-PE-Cy7 (PK-136, Biolegend #108714, 1:100 dilution), anti-CD4-APC-Cy7 (GK1.5, Biolegend #100414, 1:100 dilution), and Foxp3+ cells were identified using the GFP reporter expressed under the Foxp3 locus in Foxp3-DTR mice.

Tumor, TdLN and blood samples in Figures 5F-G and S7 were stained in 100µL with anti-CD45-BUV395 (30-F11, BD Biosciences #564279, 1:100 dilution), anti-CD4-BUV563 (RM4-4, BD Biosciences #741218, 1:100 dilution), anti-CD44-BUV737 (1M7 BD Biosciences #612799, 1:100 dilution), anti-Ki67-BV421 (16A8, Biolegend #652411, 1:100 dilution), anti-CD3-BV711 (17A2, Biolegend #100241, 1:100 dilution), anti-CD8a-FITC (53-6.7, Biolegend #100706, 1:100 dilution) anti-Foxp3-PE (FJK-16s, Invitrogen, 1:200 dilution), anti-CD25-PE-Cy5 (PC61, Biolegend #102010, 1:100 dilution), anti-NK1.1-PE-Cy7 (PK-136126, Biolegend #108714, 1:100 dilution), anti-CD62L-APC (MEL-14, Biolegend #104412, 1:100 dilution), anti-CD107a-APC-Cy7 (1D4B, Biolegend #121616, 1:100 dilution).

Tumor studies: TA99, anti-CTLA-4 (9D9), and anti-4-1BB (both with and without LAIR fusion, LOB12.3) were expressed and purified in house. anti-CD4 (GK1.5) and anti-GM-CSF (MP122E9) were purchased from BioXCell.

ELISA: goat anti-mouse IgG1 (HRP) was purchased from abcam (ab98693).

Surface binding assay: rat anti mouse IgG1-APC was purchased from Invitrogen (17-4015-82).

Validation

All commercial antibodies were validated by manufacturer and/or prior studies (Biolegend, BD Biosciences, Invitrogen, BioXCell, Abcam). Details can be found at the following links:
anti-CD45-BUV395 (30-F11, BD Biosciences #564279): https://www.bdbiosciences.com/en-us/products/reagents/flow-cytometry-reagents/research-reagents/single-color-antibodies-ruo/buv395-rat-anti-mouse-cd45.564279
anti-CD4-BUV563 (RM4-4, BD Biosciences #741218): https://www.bdbiosciences.com/en-us/products/reagents/flow-cytometry-reagents/research-reagents/single-color-antibodies-ruo/buv563-rat-anti-mouse-cd4.741218
anti-CD8α-BUV737 (53-6.7 BD Biosciences #612759): https://www.bdbiosciences.com/en-us/products/reagents/flow-cytometry-reagents/research-reagents/single-color-antibodies-ruo/buv737-rat-anti-mouse-cd8a.612759
anti-CD62L-BUV805 (MEL-14, BD Biosciences #741924): https://www.bdbiosciences.com/en-us/products/reagents/flow-cytometry-reagents/research-reagents/single-color-antibodies-ruo/buv805-rat-anti-mouse-cd62l.741924
anti-CD44-BV421 (1M7, Biolegend #103040): https://www.biolegend.com/en-us/products/brilliant-violet-421-anti-mouse-human-cd44-antibody-7225?GroupID=BLG5925
anti-Ki67-BV605 (16A8, Biolegend #652413): https://www.biolegend.com/en-us/products/brilliant-violet-605-anti-mouse-ki-67-antibody-8983
anti-CD3-BV711 (17A2, Biolegend #100241): https://www.biolegend.com/en-us/products/brilliant-violet-711-anti-mouse-cd3-antibody-10022
anti-TIM-3-BV785 (RMT3-23, Biolegend #119725): https://www.biolegend.com/en-us/products/brilliant-violet-785-anti-mouse-cd366-tim-3-antibody-14928
anti-TCF1/TCF7-AF488 (C63D9, Cell Signaling Technology #6444): https://www.cellsignal.com/products/antibody-conjugates/tcf1-tcf7-c63d9-rabbit-mab-alexa-fluor-488-conjugate/6444
anti-PD-1-PerCp/Cy5.5 (29F.1A12, Biolegend #135208): https://www.biolegend.com/en-us/products/percp-cyanine5-5-anti-mouse-cd279-pd-1-antibody-6496?GroupID=BLG7928
anti-Foxp3-PE (FJK-16s, Invitrogen #12-5773-82): https://www.thermofisher.com/antibody/product/FOXP3-Antibody-clone-FJK-16s-Monoclonal/12-5773-82
anti-CD25-PE-Cy5 (PC61, Biolegend #102010): https://www.biolegend.com/en-us/products/pe-cyanine5-anti-mouse-cd25-antibody-425?GroupID=BLG10428
anti-NK1.1-PE-Cy7 (PK-136126, Biolegend #108714): https://www.biolegend.com/en-us/products/pe-cyanine7-anti-mouse-nk-1-1-

antibody-2840?GroupID=GROUP20
anti-4-1BB-APC (17B5, Biolegend #106110): https://www.biolegend.com/en-us/products/apc-anti-mouse-cd137-antibody-10352
anti-CD3-BV785 (17A2, Biolegend #100232): https://www.biolegend.com/en-us/products/brilliant-violet-785-anti-mouse-cd3-antibody-7953
anti-CD4-APC-Cy7 (GK1.5, Biolegend #100414): https://www.biolegend.com/en-us/products/apc-cyanine7-anti-mouse-cd4-antibody-1964
anti-CD44-BUV737 (1M7 BD Biosciences #612799): https://www.bdbiosciences.com/en-us/products/reagents/flow-cytometry-reagents/research-reagents/single-color-antibodies-ruo/buv737-rat-anti-mouse-cd44.612799
anti-Ki67-BV421 (16A8, Biolegend #652411): https://www.biolegend.com/en-us/products/brilliant-violet-421-anti-mouse-ki-67-antibody-8982
anti-CD8a-FITC (53-6.7, Biolegend #100706): https://www.biolegend.com/en-gb/products/fitc-anti-mouse-cd8a-antibody-153?GroupID=BLG2559
anti-CD62L-APC (MEL-14, Biolegend #104412): https://www.biolegend.com/en-us/products/apc-anti-mouse-cd62l-antibody-381?GroupID=BLG10670

anti-CD4 (GK1.5): https://bioxcell.com/invivomab-anti-mouse-cd4-be0003-1
anti-GM-CSF (MP122E9): https://bioxcell.com/invivomab-anti-mouse-gm-csf-be0259

goat anti-mouse IgG1 (HRP, abcam, ab98693): https://www.abcam.com/products/secondary-antibodies/goat-mouse-igg1-hrp-preadsorbed-ab98693.html

rat anti mouse IgG1-APC (Invitrogen, 17-4015-82): https://www.thermofisher.com/antibody/product/Rat-anti-Mouse-IgG1-Secondary-Antibody-clone-M1-14D12-Monoclonal/17-4015-82

TA99 was synthesized in house and validated by a flow cytometry B16F10 binding assay and previous publications from our group (Agarwal et al., Nat. Biomed. Eng. 6, 129-143 (2022), Momin et al., Sci. Trans. Med. 11, eaaw2614 (2019), Zhu et al., Cancer Cell. 13, 27(4), 489-501 (2015)).

LOB12.3 (With and without LAIR fusion) was synthesized in house and validated with a flow cytometry HEK (transiently transfected to express murine 4-1BB) binding assay and an in vitro T cell activation assay. This data can be found in Fig. S1.

9D9 was synthesized in house and validated by both ELISA and flow cytometry HEK (transiently transfected to express murine CTLA-4) binding assay as detailed in Lax et al., Proc. Natl. Acad. Sci. USA. 120 (31), e2300895120 (2023).

2.5F-Fc was synthesized in house and validated by ELISA as detailed in Kwon et al., J Exp Med. 214 (6), 1679-1690 (2017).

Eukaryotic cell lines

Policy information about cell lines and Sex and Gender in Research

Cell line source(s)

B16F10 cells were purchased from ATCC (CRL-6475). Apigmented B16F10 cells used for imaging were generated by genetic deletion of Tyrosinase-related-protein-2 (TRP2), referred to as B16F10-Trp2 KO cells (as previously described in Moynihan et al., Nat. Med. 22 1402-1410 (2016)). MC38 cells were a gift from J. Schlom, National Cancer Institute, Bethesda, MD. The sex of the mouse used to generate B16F10 cells is unknown (according to manufacturer ATCC), while the MC38 tumor cell line was generated using female C57Bl/6 mice (Corbett et al., Can Res (1975)). Tumor cells were cultured in Dulbecco's Modified Eagle Medium (DMEM, ATCC) supplemented with 10% Fetal Bovine Serum (FBS, Gibco). FreeStyle 293-F cells and Expi293 cells were purchased from Invitrogen (R79007 and A14527, respectively) and cultured in FreeStyle expression medium (Gibco) and Expi293 expression medium (Gibco), respectively. CHO DG44 cells were a gift from David Hacker and cultured in ProCHO5 (Lonza) supplemented with 4 mM L-glutamine, 0.1 mM hypoxanthine, and 16 µM thymidine. Tumor cells were maintained at 37°C and 5% CO2 and FreeStyle 293-F cells, Expi293 cells, and CHO DG44 cells were maintained at 37°C and 8% CO2.

Authentication

All cell lines were maintained separately and frozen down after early passage number to ensure correct identity and minimize risk of contamination. No further in-house authentication was performed after procuring cell lines.

Mycoplasma contamination

All cell lines were confirmed free of mycoplasma by PCR

Commonly misidentified lines
(See ICLAC register)

No commonly misidentified cell lines were used in this study

Animals and other research organisms

Policy information about studies involving animals; ARRIVE guidelines recommended for reporting animal research, and Sex and Gender in Research

Laboratory animals

C57Bl/6 (C57Bl/6NTac, B6-F) mice were purchased from Taconic. C57Bl/6 albino (B6(Cg)-Tyrc-2J/J, #000058) mice were purchased from The Jackson Laboratory. C57Bl/6 Foxp3-DTR(B6.129(Cg)-Foxp3tm3(DTR/GFP)Ayr/J, #016958) mice were a gift from the Spranger lab (MIT). C67Bl/6 OT-I mice (C57BL/6-Tg(TcraTcrb)1100Mjb/J, #003831) were a gift from the Irvine lab (MIT). B6 Foxp3-DTR mice and B6 OT-I mice were bred in house and genotyped using Transnetyx. All mice used were between the ages of 6 and 12 weeks old. Mice were housed in a specific-pathogen free facility and fed normal chow and water ad libitum under standard animal facility conditions (12 hour light/dark cycle, temperature of 22 C, relative humidity of 40%-70%.

Wild animals

No wild animals were used in this study

Reporting on sex	All studies were conducted using female mice, consistent with prior studies in our lab (Lax et al., Proc. Natl. Acad. Sci. USA. 120 (31), e2300895120 (2023), Lutz et al., Proc. Natl. Acad. Sci. USA. 119 (36), e2205983119 (2022), Agarwal et al., Nat. Biomed. Eng. 6, 129-143 (2022), Momin et al., Nat. Commun. 13, 109 (2022), Mehta et al., Nat. Biomed. Eng. 4, 636-648 (2020), Momin et al., Sci. Trans. Med. 11, eaaw2614 (2019).
Field-collected samples	No field collected samples were used in this study
Ethics oversight	All animal work was conducted under the approval of the Massachusetts Institute of Technology Committee on Animal Care in accordance with federal, state, and local guidelines.

Note that full information on the approval of the study protocol must also be provided in the manuscript.

Plants

Seed stocks	N/A
Novel plant genotypes	N/A
Authentication	N/A

Flow Cytometry

Plots

Confirm that:

☒ The axis labels state the marker and fluorochrome used (e.g. CD4-FITC).

☒ The axis scales are clearly visible. Include numbers along axes only for bottom left plot of group (a 'group' is an analysis of identical markers).

☒ All plots are contour plots with outliers or pseudocolor plots.

☒ A numerical value for number of cells or percentage (with statistics) is provided.

Methodology

Sample preparation	Tumors were excised, weighed, and mechanically dissociated before being enzymatically digested using a gentleMACS Octo Dissociator with Heaters (Miltenyi Biotec) in gentleMACS C tubes (Miltenyi Biotec) and enzymes from the Mouse Tumor Dissociation Kit (Miltenyi Biotec). Tumors were digested using the 37C_m_TDK_1 program for soft tumors. Following digestion, tumors were filtered through a 40 µm filter and transferred to a V-bottom 96 well plate for staining. TdLN and spleens were excised, weighed, and mechanically dissociated through a 70 µm filter. Spleen samples were resuspended with 5 mL of ACK Lysis buffer (Gibco) to lyse red blood cells before being re-filtered through a 70 µm filter. TdLN and spleen samples were then transferred to a V-bottom 96 well plate for staining. Blood samples were collected via cardiac puncture into K3 EDTA coated tubes (MiniCollect). 200 µL of blood was mixed with 1 mL of ACK lysis buffer (Gibco) to lyse red blood cells before being transferred to a V-bottom 96 well plate for staining. Precision Counting Beads (Biolegend) were added to each well to account for sample loss during processing and obtain accurate counts. Cells were then stained for relevant markers and analyzed according to the methods outlined in the manuscript.
Instrument	Cells were analyzed on a BD FACS Symphony A3 (BD Biosciences) analyzer.
Software	BD FACSDiva (BD Biosciences v7) was used for the collection of flow cytometry data, and FlowJo (v10) was used for analysis. The collected data were plotted with statistical analysis by GraphPad Prism (v10).
Cell population abundance	No sorting was performed in this study.
Gating strategy	Example gates used to identify cell populations are shown in Supplementary Figure 10 and examples of phenotypic gating is shown in relevant figures. Briefly, cells were gated on FSC-A vs. SSC-A to exclude debris and then FSC-H vs. FSC-W followed by SSC-H vs. SSC-W to exclude doublets. Cells were then fated on Live/Dead vs. FSC-A to exclude dead cells and then CD45 vs. FSC-A to identify immune cells. T cells and NK cells were next identified by CD3 vs. NK1.1 and T cells were subsequently gated on CD4 vs. CD8a. Finally, CD4 T cells were gated further to identify Foxp3+ CD25+ double positive Tregs (or in the case of Foxp3-DTR mice, the Foxp3 GFP reporter was used to identify Tregs). For phenotypic markers, example gating can be found in main text and/or supplementary figures for each marker.

☒ Tick this box to confirm that a figure exemplifying the gating strategy is provided in the Supplementary Information.
